# Supplementary material for: Properties of Multidrug-Resistant Mutants Derived from Heterologous Expression Chassis Strain Streptomyces albidoflavus J1074
Source: Microorganisms. 2023 Apr 30;11(5):1176. doi: 10.3390/microorganisms11051176 (PMC10220713; doi:10.3390/microorganisms11051176)
Supplement: Supplementary file 1 [file microorganisms-11-01176-s001.zip › microorganisms-2322696-supplementary.pdf]

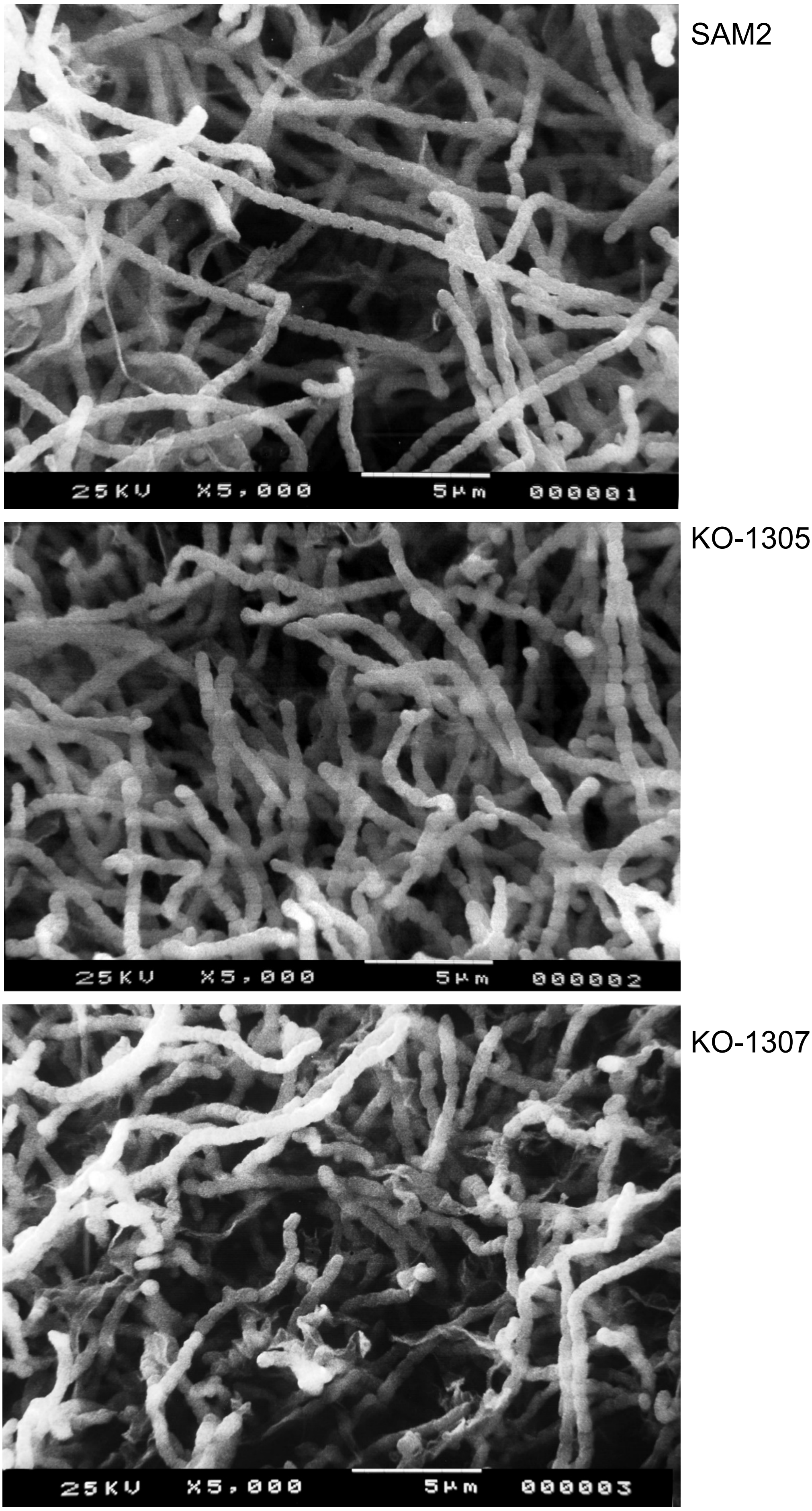

Figure S1. Scanning electron microscopy of lawns of *S. albidoflavus* KO-1305 and KO-1307 strains. The strains (as well as parental SAM2) were grown on SFM agar for five days, and then processed as described in https://pubmed.ncbi.nlm.nih.gov/31017319/

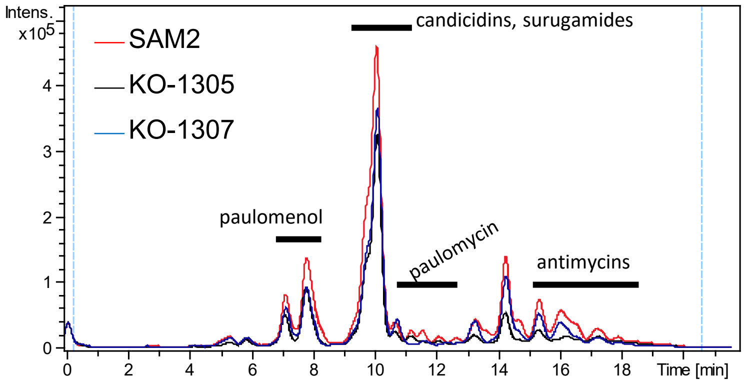

Figure S2. Overlaid MS traces (negative ionization) of ethyl acetate extracts from strains SAM2, KO-1305, KO-1307 grown in medium R5S for 120 h. The traces are normalized to equal amounts of the biomass. Major fractions corresponding to known natural products of *S. albidoflavus* are labeled with thick horizontal lines and annotated above. The chromatogram reflect typical result of three biological repeats. More details are below in Figures S5-S9.

Table S1. Mutations and rifampicin resistance levels of various of *rpoB* mutants isolated from *S. albus* KO-1305 and KO-1307

Strain	Mutation in rpoB	Amino acid substitution*	Mutant frequency**	Rif resistance (µg/mL)
**J1074**	-	-	-	3
**KO-1305▼**	-	-	-	3
**KO-1401**	1325C→T	Ser442→Leu	1/48	30
**KO-1402**	1262C-1273T→Δ	Q421-F425→L421	1/48	200
**KO-1403**	1298G→C	Ser433→Trp	1/48	10
**KO-1404**	1310A→G	His437→Arg	13/48	>200
**KO-1405**	1309C→T	His437→Tyr	15/48	200
**KO-1406**	1318C→A	Arg440→Ser	1/48	20
**KO-1407**	1318C→T	Arg440→Cys	5/48	25
**KO-1408**	1319G→A	Arg440→His	7/48	25
**KO-1409**	1271A→T	Gln424→Leu	2/48	200
**KO-1307▼**	-	-	-	3
**KO-1410**	1325C→T	Ser433→Leu	1/37	25
**KO-1411**	1310A→G	His437→Arg	12/37	>200
**KO-1412**	1451C→T	Pro475→Leu	1/37	25
**KO-1413**	1309C→T	His437→Tyr	16/37	200
**KO-1414**	1309C→G	His437→Asp	3/37	100
**KO-1415**	1271A→T	Gln424→Leu	1/37	50
**KO-1416**	1310A→C	His437→Pro	1/37	>200
**KO-1417**	1319G→A	Arg440→His	1/37	25

***Highlighted cells correspond to substitutions that are, to the best of our knowledge, observed for the first time in Streptomyces (see also Fig. S10)**
****Number of mutants out of total Rif^r clones isolated from a given KO strain**
▼Parental strain for respective Rif^r lineages

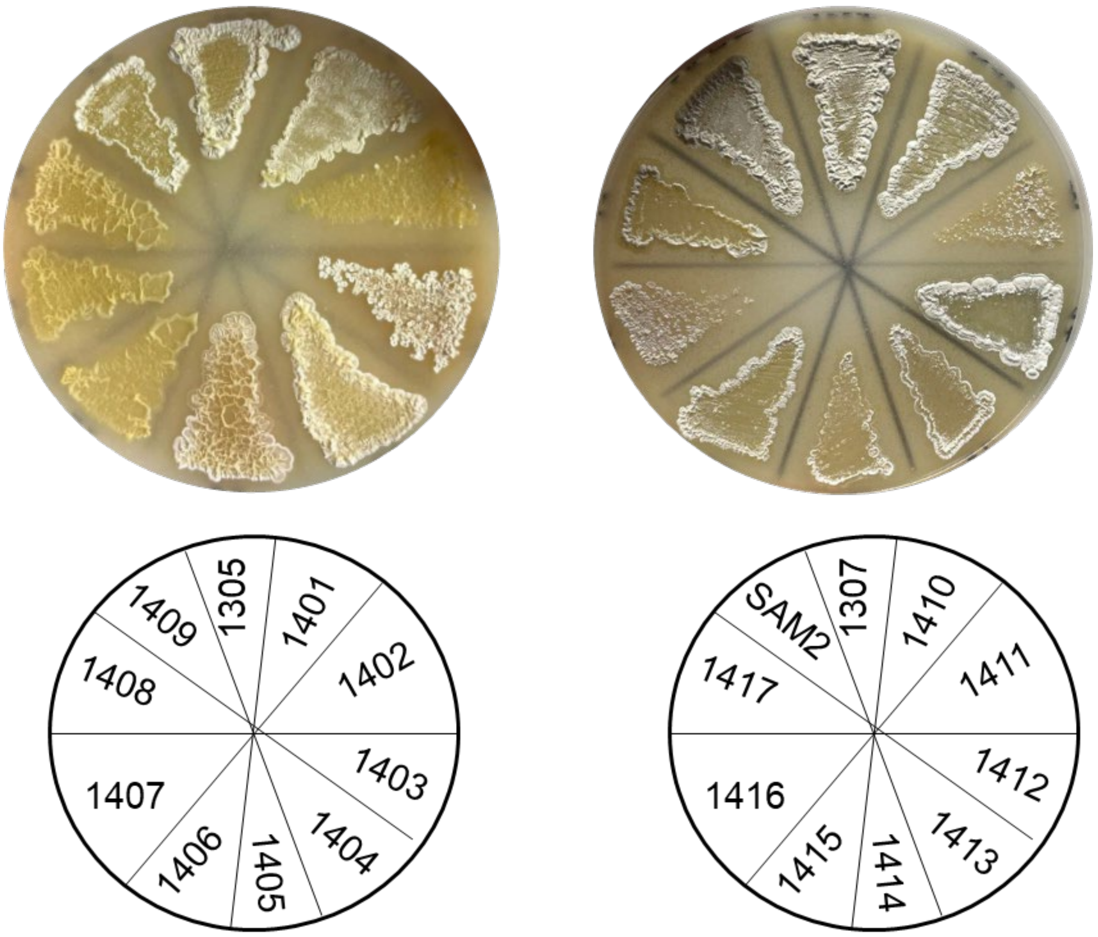

Figure S3. Lawns of KO-1401 to KO-1417 strains on soy flour mannitol agar. The lawns were grown for five days, please note the absence of whitish aerial mycelium in strains KO1402, 1406-1408. Please also note that KO-1410 to KO-1417 strains based on *rpsL* merodiploid R94G_ex are less affected in the morphology.

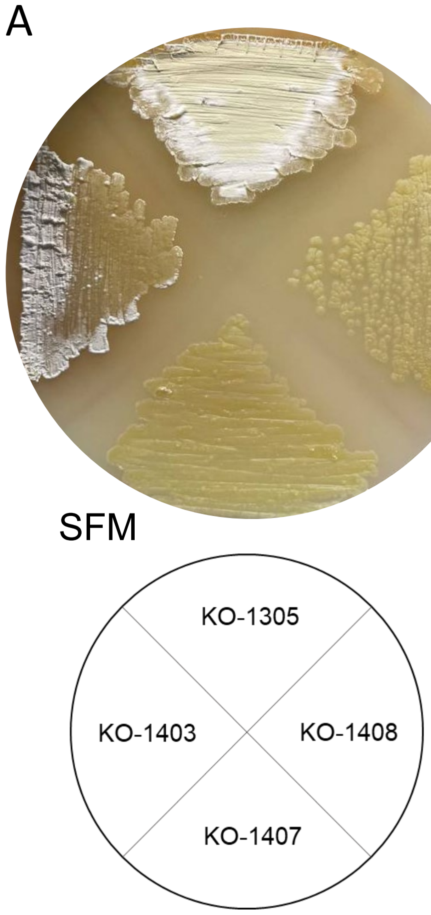

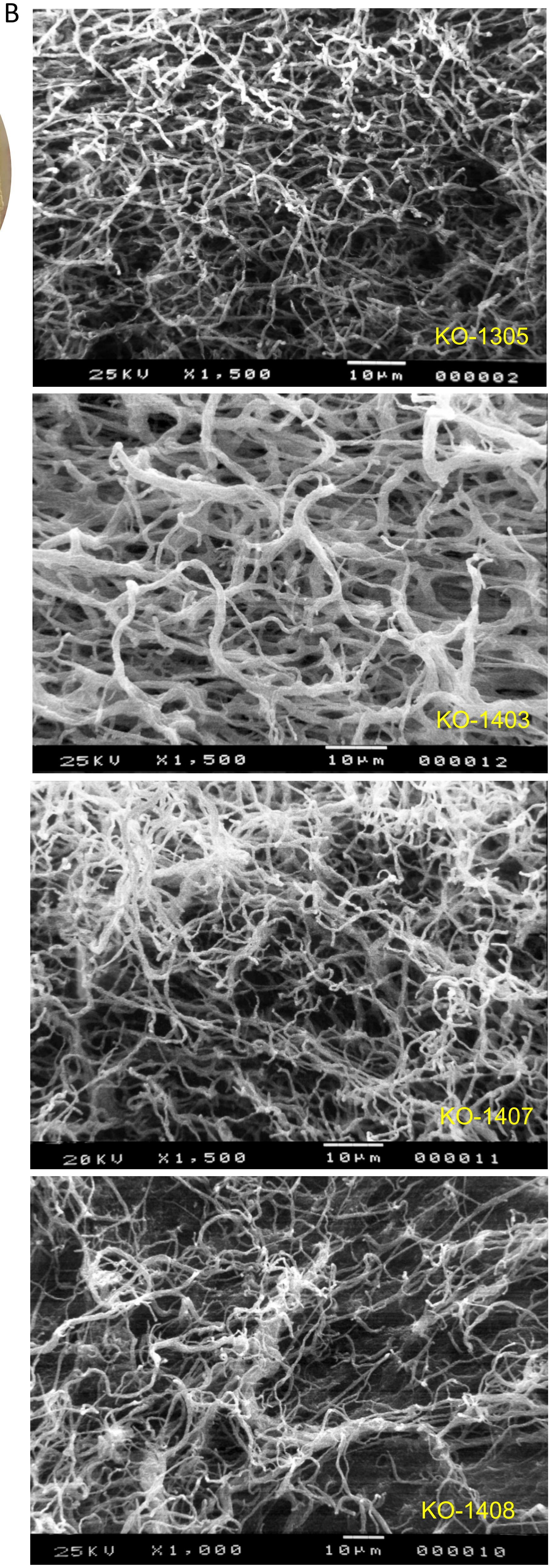

Figure S4. Morphology of solid cultures of selected Rif^r strains. **A.** Lawns of KO-1305, KO-1403, 1407 and KO-1408 strains on soy flour mannitol agar after 120 h of growth. **B.** Scanning electron microscopy of the same lawns. KO-1305, KO-1403, KO-1407 and KO-1408 strains.

Table S2. Susceptibility of selected Rif^r strains of *S. albidoflavus* to beta-lactams, ciprofloxacin, tetracycline and kanamycin

Strain	Growth inhibition zones (mm)				
	Imipenem*	Cefalexin*	Ciprofloxacin*	Tetracycline*	Kanamycin*
**SAM2**	26±7	14±4	34±1	28±1	24±3
**KO-1305**▼	29±8	13±3	37±1	31±1	23±1
**KO-1403**	30±8	15±3	37±4	30±1	23±1
**KO-1406**	30±6	22±5	42±0**	43±3	25±2
**KO-1407**	32±7	20±3	45±3	41±1	24±2
**KO-1408**	21±5	12±0	43±3	41±1	23±1
**KO-1307**▼	27±5	14±2	33±3	34	26
**KO-1412**	31±4	19±4	38±1	32±1	23±1
**KO-1414**	27±5	0±0	42±2	36±1	23±2

▼Parental strain for respective Rif^r lineages

*Amount of antibiotic per disk, in μg: imipenem – 10; cefalexin – 30; ciprofloxacin – 5; tetracycline – 30; kanamycin – 10

**Values on yellow background are significantly different from the parental strains (no overlap between variations in the diameters of growth inhibition zones of the respective entries of the parental and mutant strains) . See the example of disk diffusion assay below

KO-1406 SAM2 KO-1414

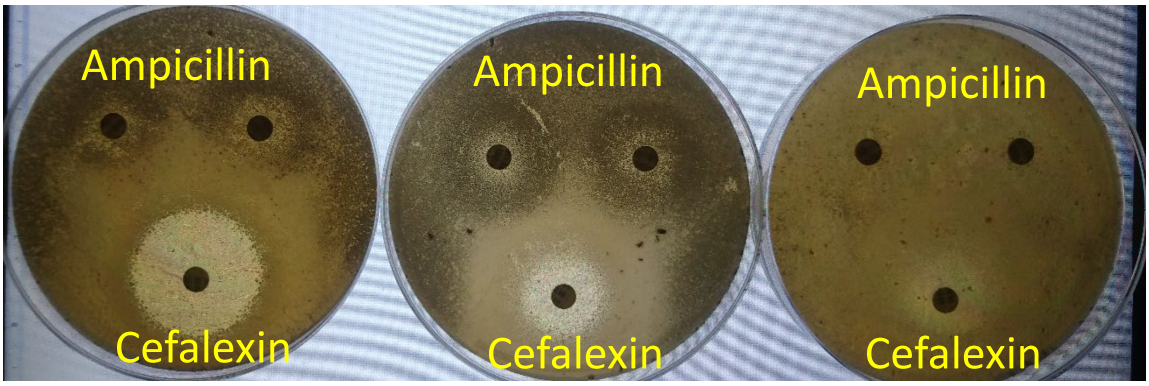

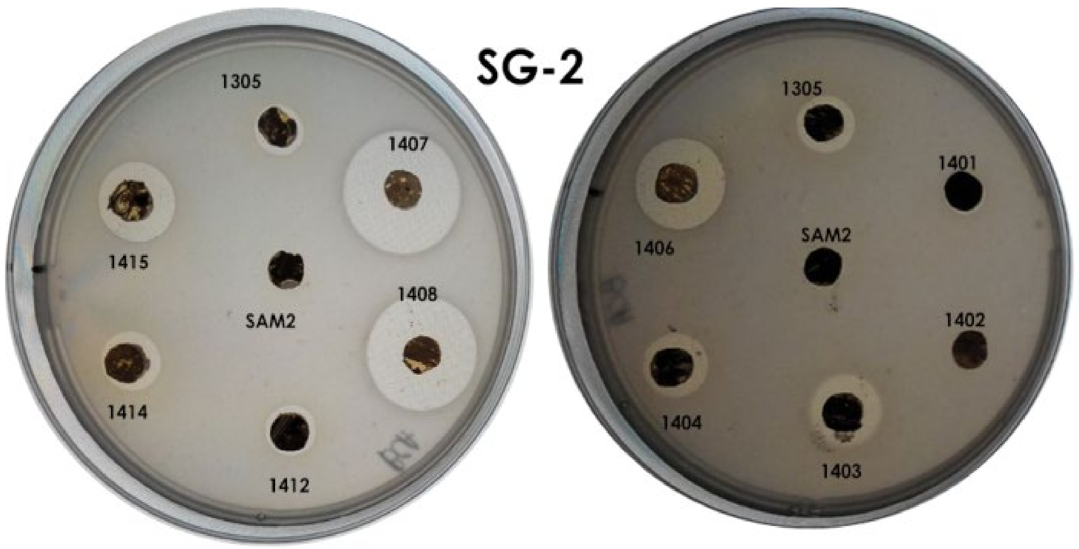

Figure S5. An example of agar plug assay of antibiotic activity of *rpoB* mutants. For details, please see the main text.

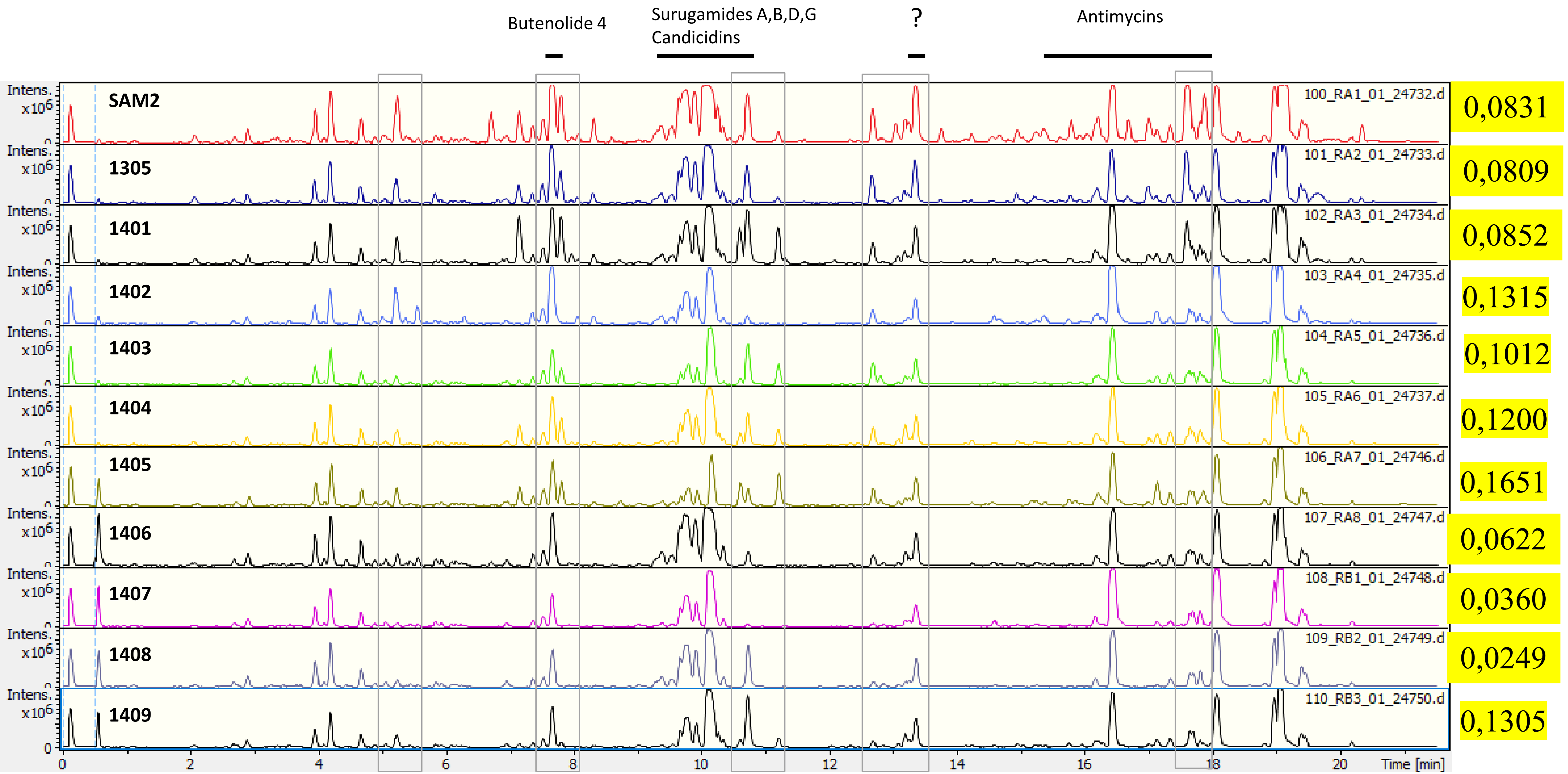

Figure S6. Stacked MS traces (positive ionization mode) for KO-1305 and its Rif^{r} derivatives. These MS traces are not normalized to equal amounts of the biomass; the latter (in g/10 mL of fermentation medium) is marked to the right of the chromatogram (yellow background). Thick black lines above the chromatograms denote major knonw classes of small molecules produced by J1074. Mass peaks present in unknown fraction (labeled with interrogation sign) is detailed below in Figure S9.

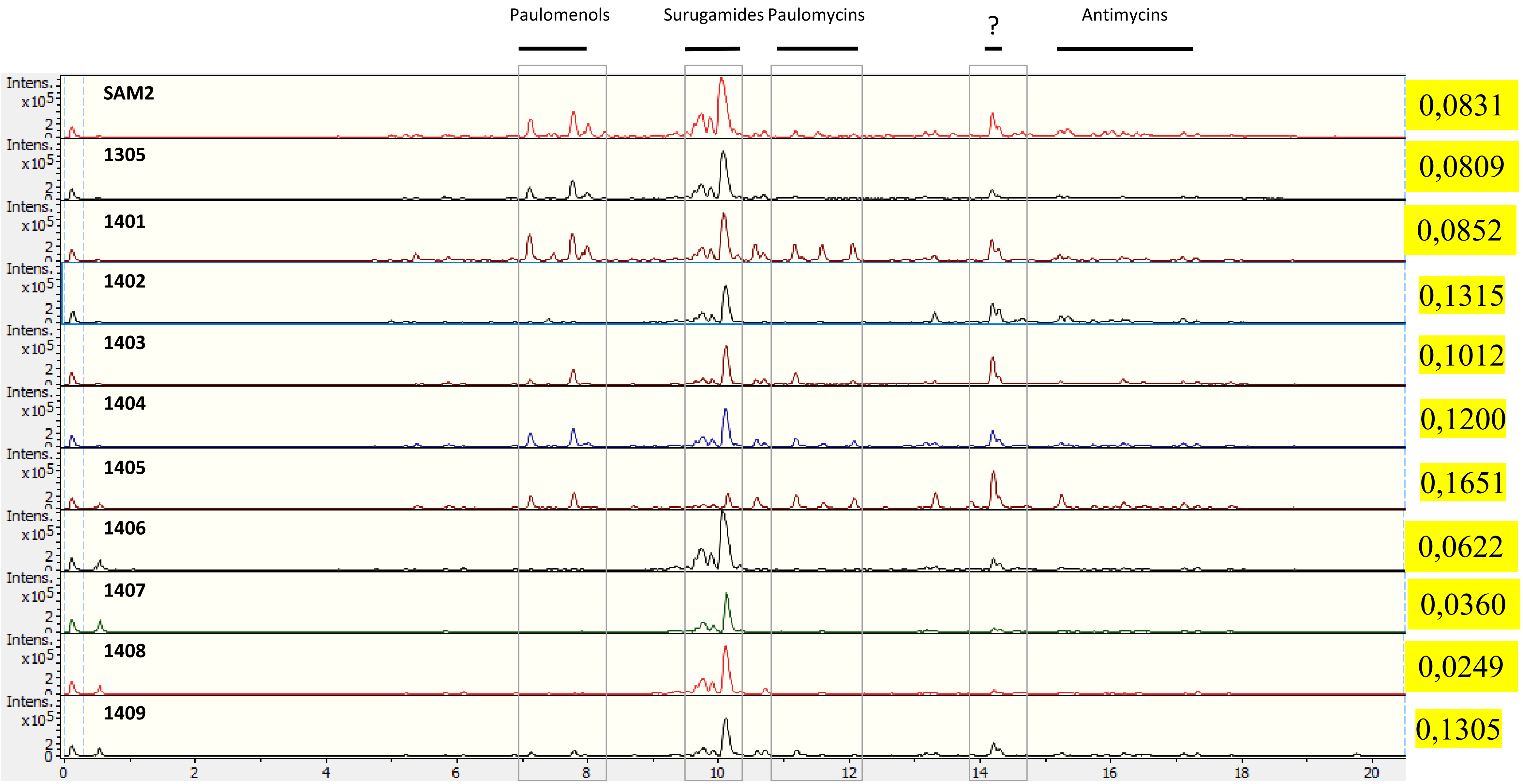

Figure S7. Stacked MS traces (negative ionization mode) for KO-1305 and its Rif^r derivatives. These MS traces are not normalized to equal amounts of the biomass; the latter (in g/10 mL of fermentation medium) is marked to the right of the chromatogram (yellow background). Thick black lines above the chromatograms denote major knonw classes of small molecules produced by J1074. Mass peaks present in unknown fraction (labeled with interrogation sign) is detailed below in Figure S9.

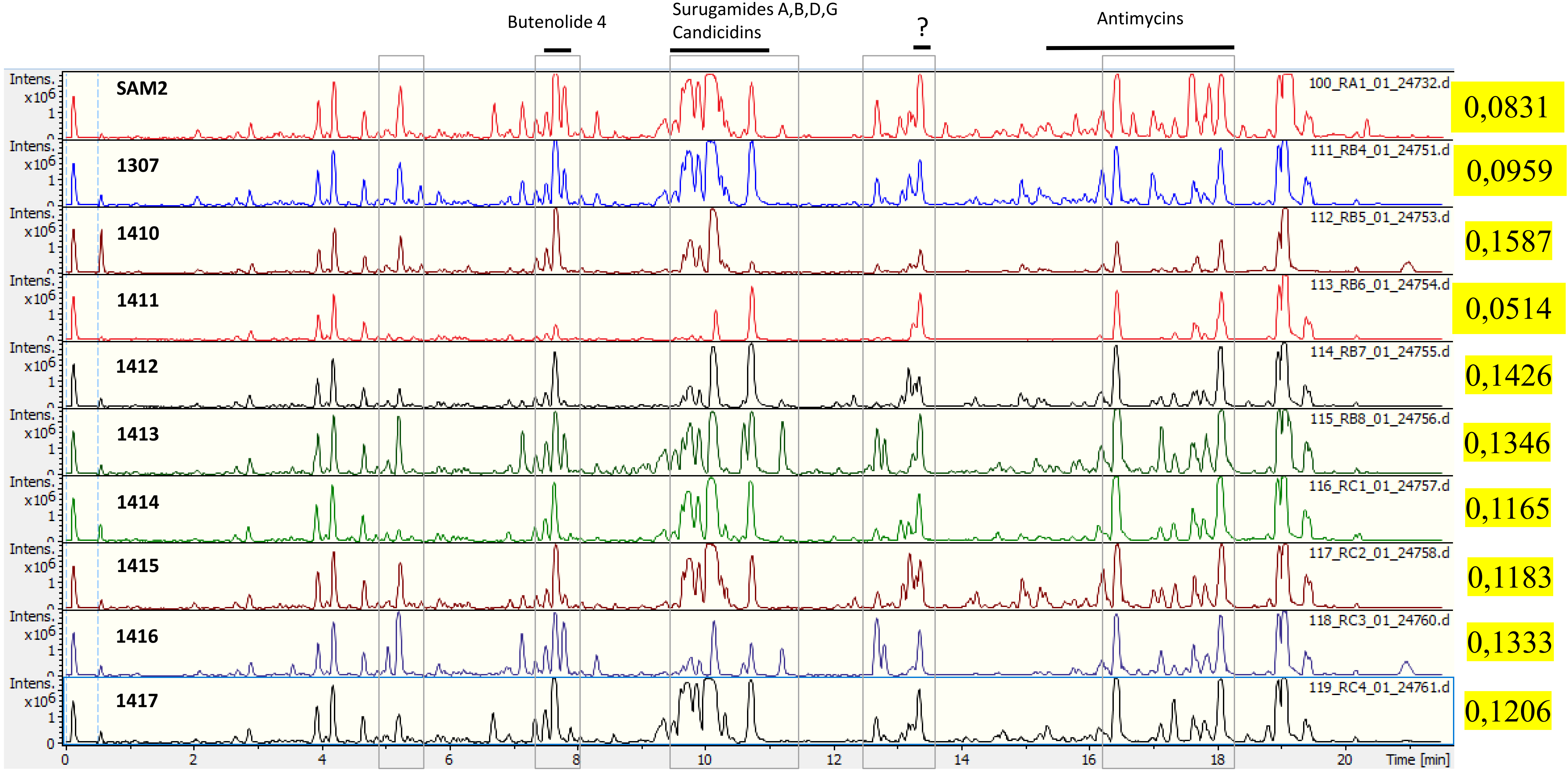

Figure S8. Stacked MS traces (positive ionization mode) for KO-1307 and its Rif^r derivatives. These MS traces are not normalized to equal amounts of the biomass; the latter (in g/10 mL of fermentation medium) is marked to the right of the chromatogram (yellow background). Thick black lines above the chromatograms denote major knonw classes of small molecules produced by J1074. Mass peaks present in unknown fraction (labeled with interrogation sign) is detailed below in Figure S9.

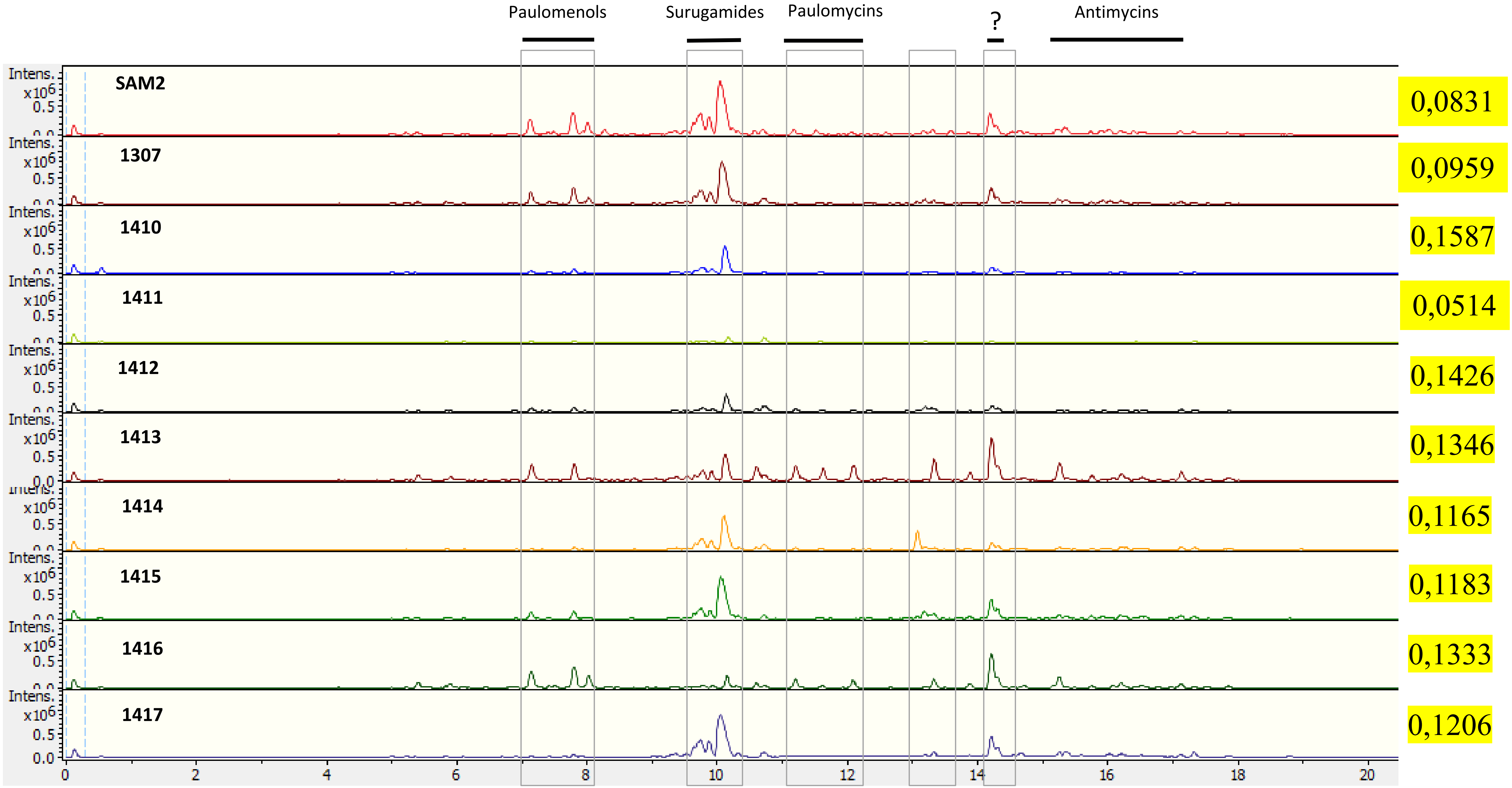

Figure S9. Stacked MS traces (negative ionization mode) for KO-1307 and its Rif^r derivatives. These MS traces are not normalized to equal amounts of the biomass; the latter (in g/10 mL of fermentation medium) is marked to the right of the chromatogram (yellow background). Thick black lines above the chromatograms denote major knonw classes of small molecules produced by J1074. Mass peaks present in unknown fraction (labeled with interrogation sign) is detailed below in Figure S9.

A

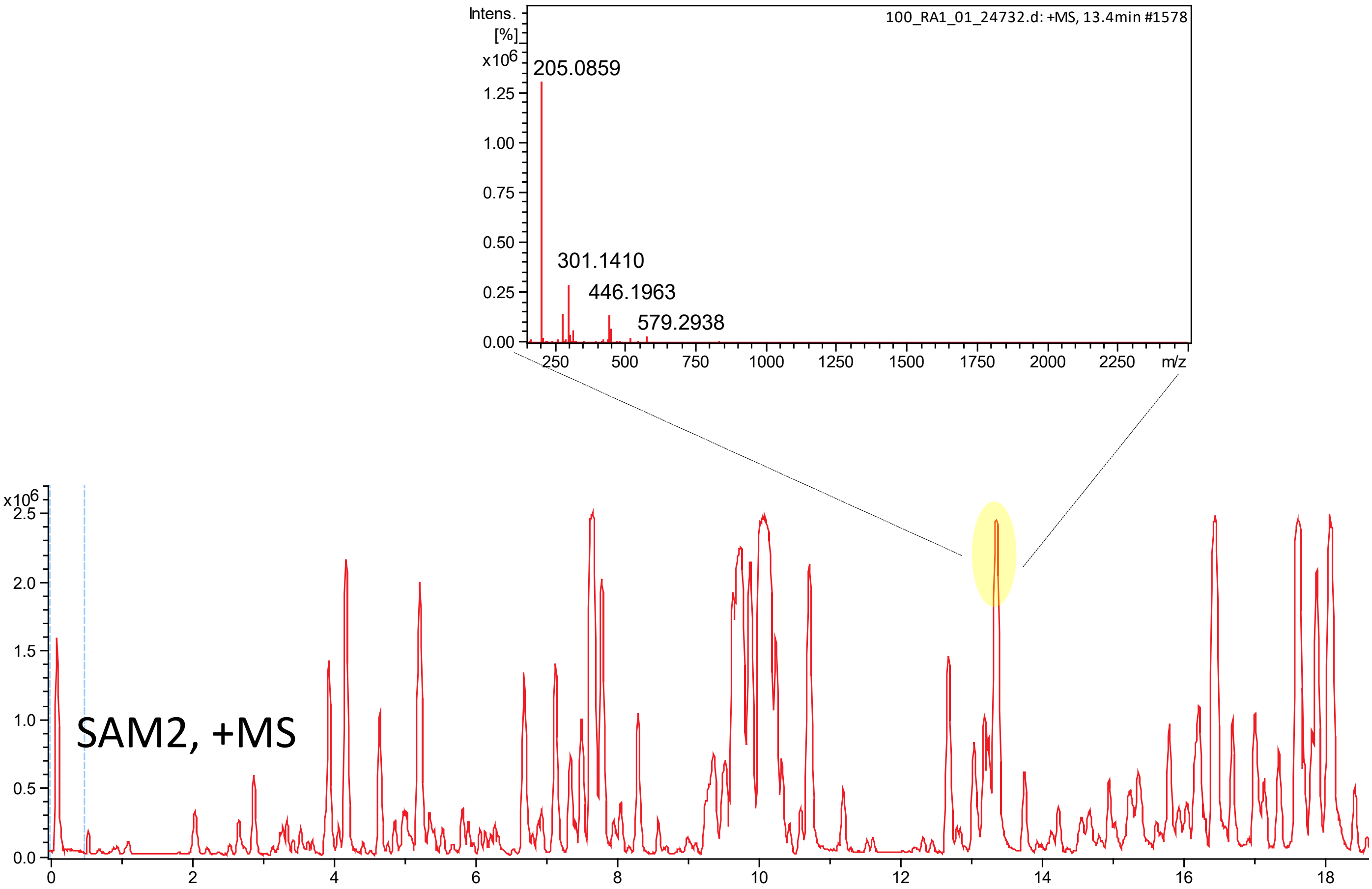

B

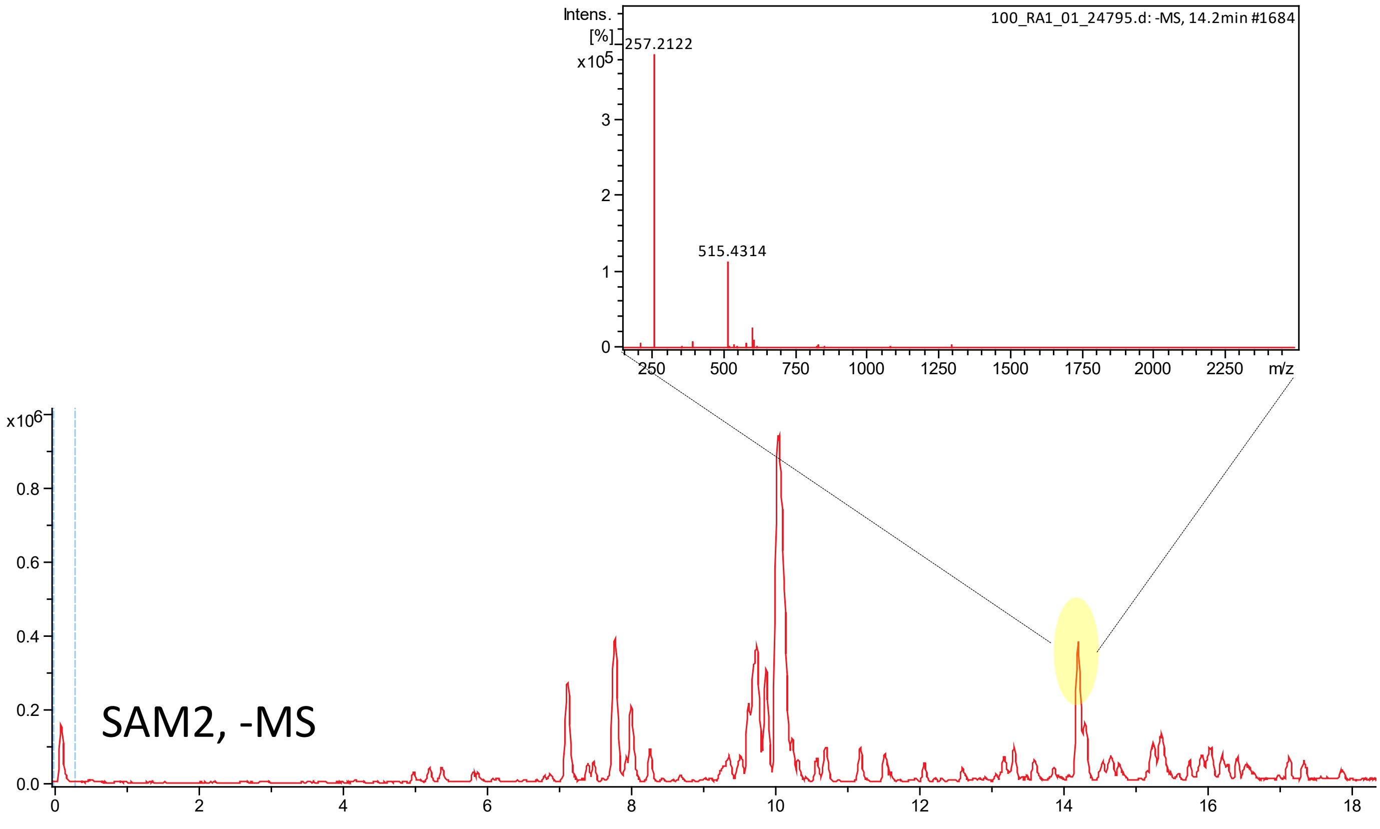

Figure S10. Mass-spectra within the range of elution of unknown fractions observed in extracts of *S. albidoflavus* strains (marked with "?" in Figures S5-S8), in positive (**A**) and negative (**B**) ionization modes. Identical mass spectra were observed in the extracts from all mutants.

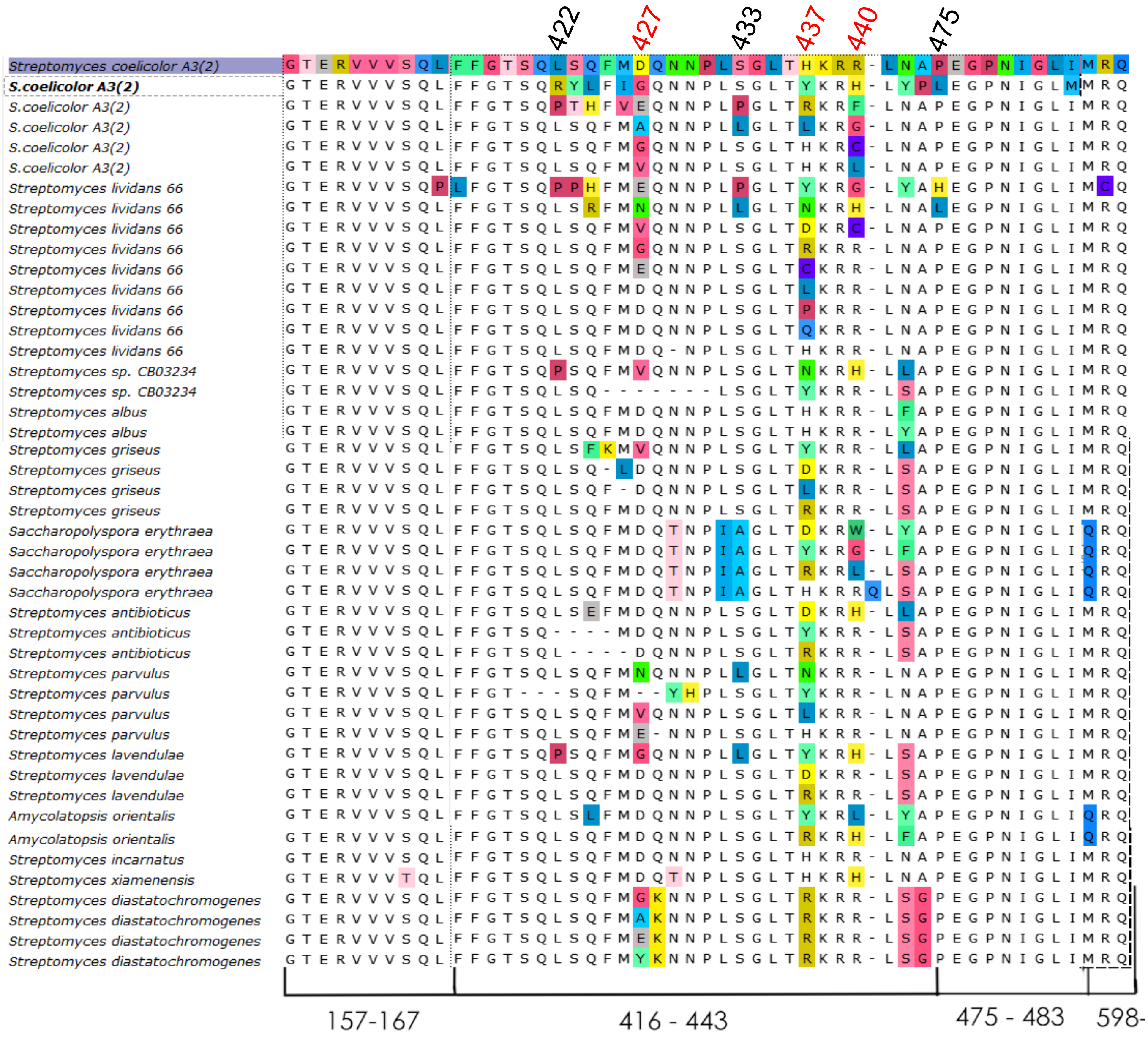

Figure S11. Diversity of *rpoB* mutations isolated in different *Streptomyces*, *Saccharopolyspora* and *Amycolatopsis* species. The multiple amino acid sequence aligment was built from four noncontiguous segments of RpoB protein (their coordinates within the entire protein are marked at the bottom of the figure, square brackets) where Rif^r-mutations occur. Top sequence, wild type RpoB protein of *S. coelicolor*. Amino acid substitutions are highlighted with color. Number above the alignment mark several amino acid positions, those in red label sites where Rif^r-conferring mutations occur most frequently. Information about *rpoB* mutations was retrieved from PubMed.

A

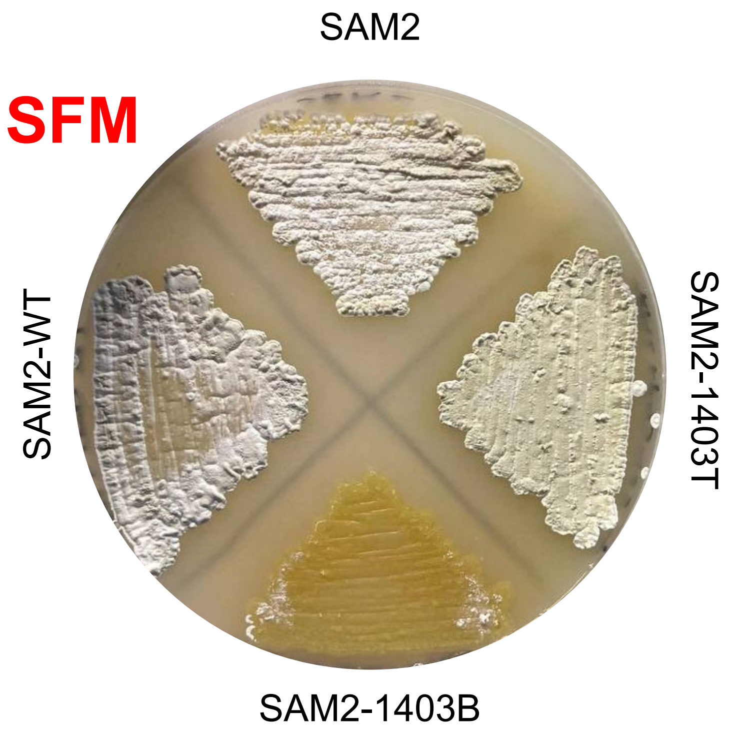

B

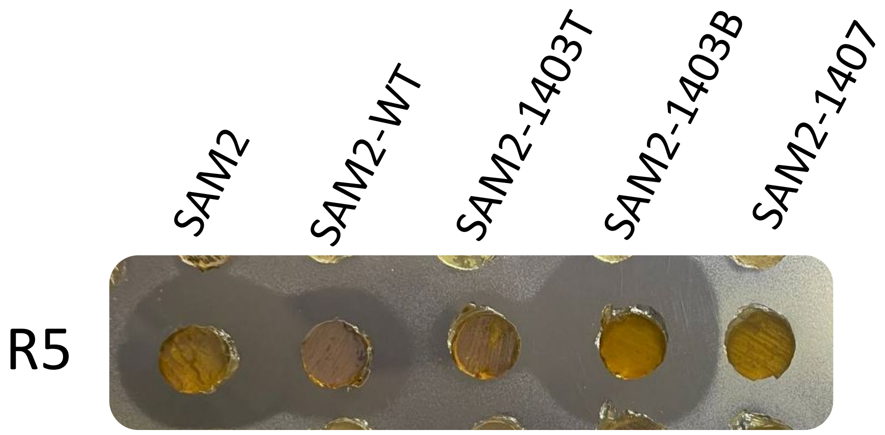

Figure S12. Morphology and antibiotic activity of *rpoB* merodiploids. A. Lawns of the strains on soy flour mannitol agar, 120 h. B. Agar plug assay of antibiotic activity of rpoB merodiploids against B. cereus. Laws were grown on R5 medium for 120 h/
